# Sociodemographic Determinants of Telehealth Visits: A Comparison of Mental Health and Substance Use Disorders with Other Services

**DOI:** 10.3390/epidemiologia6030054

**Published:** 2025-09-03

**Authors:** Stanley Nkemjika, Orman Trent Hall, Jeanette Tetrault, Ayana Jordan

**Affiliations:** 1 Department of Psychiatry and Human Behavior, Thomas Jefferson University, Philadelphia, PA 19103, USA; 2 Department of Psychiatry and Behavioral Health, Ohio State University Wexner Medical Center, Columbus, OH 43210, USA; 3 Program in Addiction Medicine, Department of Internal Medicine, Yale School of Medicine, New Haven, CT 06510, USA; 4 Department of Psychiatry, New York University Grossman School of Medicine, New York, NY 11220, USA

**Keywords:** discrimination, telehealth, mental health, race/ethnicity, sociodemographic

## Abstract

Background/Objectives: Discrimination has wide-ranging implications, affecting patients’ trust in healthcare professionals and their intentions to seek care. It can cause barriers that can affect access to care, especially among racial and ethnic minority groups in mental healthcare settings. Hence, we aim to examine the relationship between racial discrimination and reasons for using telehealth services. Method: Using the 2022 Health Information National Trends Survey (HINTS-6), we isolated 6017 participants who reported on discrimination inquiry. The primary dependent variable in this study is self-reported discrimination in healthcare, while the main independent variable is the reason for the most recent telehealth visit, and several socio-demographic variables were included as covariates, including age, sex, education, income, and marital status. Descriptive statistics were generated, and multivariable regression analysis was estimated as well, with a *p*-value < 0.05. Results: Non-Hispanic Black individuals had significantly higher odds of reporting discrimination compared to non-Hispanic White individuals (crude OR: 11.85, 95% CI: 7.67–18.309). Similarly, Hispanic individuals (crude OR: 4.626, 95% CI: 2.899–7.384) and individuals of other racial backgrounds (crude OR: 6.883, 95% CI: 4.04–11.729) had significantly increased odds of experiencing discrimination. Conclusions: The findings of this study offer critical insights into the determinants of perceived discrimination within telehealth services. Given the sustained integration of telehealth into healthcare delivery, it is imperative to develop and implement targeted strategies that provide education and resources to promote health equity among Non-Hispanic Black patients.

## 1. Introduction

Racial discrimination in healthcare systems has been well documented as a prominent barrier to care-related access [1,2,3], although significant areas of the literature remain underexplored in terms of the complete depth of these disparities. Discrimination has wide-ranging implications, affecting patients’ trust in healthcare professionals and their intentions to seek care [3,4]. There are barriers to the care faced by racial and ethnic minority groups, particularly in mental healthcare settings, due to racism and discrimination [5,6]. These factors restrict their access to health services and worsen health dynamics. Discriminatory practices in medical settings can lead to an erosion of trust, causing many individuals to avoid the care they need, which could further worsen already existing health disparities [7]. Hence, there is a need for research to examine experiences of discrimination across mental health, non-mental health, and substance use services, and its role in affecting access and outcomes of healthcare, particularly in marginalized communities.

Evidence in the literature suggests that discrimination in healthcare settings is especially evident in the area of opioid addiction treatments among Black patients, where studies show a dramatic lack of trust in healthcare workers [8,9]. Black patients in addiction treatment settings frequently report experiencing racial discrimination from healthcare providers, according to the Kaiser Family Foundation (KFF) [10], causing them to be reticent about continuing treatment. This leads to larger problems in healthcare access and adherence as patients fear they might be turned away, and then never come back. These delays may exacerbate health outcomes, which increase racial health disparities, given that patients who avoid care may find themselves much sicker in the long run [11]. Mistrust is likely a significant factor in the failure to utilize care due to the evidence previously mentioned; however, it is important to understand the mechanics of how racial discrimination can undermine not only initial engagement with a needed healthcare service, but also engender long-lasting effects on compliance with longer-term treatment as well.

Disparities in access to and quality of mental healthcare among different racial and ethnic groups only accelerate the discussion of how racial discrimination affects healthcare [12]. The systemic effects of racism on healthcare system access and health outcomes have been established [10], and the lack of diagnosis and treatment, particularly among minorities, exacerbates those established effects. These gaps in care are frequently attributed to prejudiced attitudes among healthcare providers and a lack of culturally competent care in clinical environments [13,14]. Moreover, persistent exposure to discrimination, known through the minority stress theory [15], induces high amounts of stress that ultimately may have both mental and physical health implications. These stress-related health conditions are especially concerning because they disproportionately impact communities of color, a factor compounding the challenges these communities already face in accessing timely, appropriate mental healthcare. These have all been increasingly addressed as public health and socioeconomic concerns. Still, it takes a more nuanced understanding of how systemic discrimination, in all its forms, including racism, sexism, classism, and fatphobia, impacts mental health, especially in marginalized and oppressed populations.

In addition, the link between racial discrimination and substance use behaviors adds another level of complexity to the relationship between discrimination and healthcare access. According to Gibbons et al. (2010), experiences of racial discrimination make an increase in substance use more likely, as individuals may turn to drugs and alcohol as coping mechanisms to alleviate the emotional and psychological distress associated with these negative experiences [16]. The weathering hypothesis framework [17], in which the compounding impacts of systemic bias lead to a kind of health-stacking, severely refining deterioration to African American populations, posits that systemic, socio-political, and economic stressors contribute to the early onset of health conditions, and include health disorders and substance use. In light of these “multimodal” overlapping challenges, how can healthcare systems positively impact these areas by standing against the causes of substance use across minority communities alongside discriminatory practices that aggravate disparities in health systems? Further research needs to be conducted into culturally relevant interventions that not just address substance use but also the wider social determinants stemming from discrimination in healthcare settings.

While evidence of discrimination based on race during healthcare interactions is growing, there are still considerable gaps in our understanding of its longer-term implications for health outcomes and access to healthcare. Research has demonstrated that implicit biases of healthcare providers lead to misdiagnosis, decreased compassion, and inadequate treatment regimens for racial minorities [18,19]. In addition, disparities in insurance coverage, compounded by socio-economic inequalities, also restrict access to quality healthcare services amongst racial minorities, including mental health and substance use treatment [20]. Culturally competent care, including anti-racism training for healthcare professionals and policy reforms to reduce structural racism, is key to addressing these inequities [21]. Although community-level interventions have been demonstrated to be effective in reaching out to marginalized stakeholder segments in health promotion [22], telehealth also emerged as a potential solution to reach these populations when making health more accessible [23]. But the effectiveness of these interventions must be considered under systemic racism in order to provide benefits for all populations equitably. For example, Hamnvik et al. (2022) highlighted that telemedicine challenges, such as technological literacy gaps, lack of private spaces for care, and inconsistent regulatory and reimbursement frameworks, may limit its full effectiveness for this transgender population [24]. However, research shows that racial minorities, in particular, Black, Hispanic, and Indigenous people, still encounter barriers to obtaining equitable care and experience worse health outcomes and a lower level of care quality [25]. Thus, the social gap is in knowing how such racial discrimination experiences further differ between the sectors of healthcare services (e.g., general medical care, mental health services, and addiction services) [10,18]. Irrespective of systemic racism and institutional biases, the intersection of race with other social determinants such as socioeconomic background, gender, and sexual orientation, provides further layers of complexity to the issue that have received scant exploration in the empirical literature to date [26,27]. As such, the need for more focused research is emerging based on the existing literature, where a majority of studies have focused on single forms of discrimination, while the intersectional, multiple types of discrimination have received scant attention.

Another important factor to consider is the connection between racial discrimination and access to healthcare services, which is a key determinant of public health. This is also crucial for developing effective policies to implement a functional healthcare system in a relatively short time frame. Evidence in the literature has demonstrated that the individuals from racial minority groups experience dramatically higher levels of discrimination in healthcare contexts, resulting in lower trust, evasiveness of care, and postponement of care, which consequently contributes to growing health disparities [16,17]. As the United States population continues to diversify, and the various healthcare outcomes associated with discrimination along the lines of race and ethnicity generate concern, targeted research is warranted to better understand how these factors converge within, across, and along different varieties of care (physical health, mental health, substance use, and so on) [28,29]. Hence, we need to know and understand more about the role of race-based obstructed access to healthcare services operates before we can proffer an intervention plan. We hypothesize that there is a significant difference in the likelihood of reporting racial discrimination in healthcare based on the category of health-related reason for the most recent telemedicine use. Thus, the primary objective of this study is to examine the association between racial discrimination and the reasons for using telehealth services.

## 2. Methods

### 2.1. Study Design

This study employs a quantitative, cross-sectional design, utilizing secondary data from the Health Information National Trends Survey (HINTS-6) collected by the National Cancer Institute (NCI). The survey was designed with robust statistical methodologies and reliable sampling techniques to ensure comprehensive and representative data [30]. HINTS-6 includes a variety of questions on telehealth usage, experiences of discrimination in healthcare, and broader social and health-related factors. This dataset is particularly valuable for investigating the relationship between telehealth visit reasons and discrimination, especially among vulnerable populations [30]. Secondary data analysis was conducted using existing responses from the HINTS-6 survey, with no new participants recruited for this study.

### 2.2. Study Population

HINTS-6 is a nationally representative survey of civilian, non-institutionalized adults aged 18 and older living in the United States. The 2022 HINTS-6 survey aimed to gather data from 7000 completed questionnaires to capture a broad and diverse sample. The survey uses a two-stage sampling design, with the first stage involving the random selection of residential addresses from across the U.S., ensuring a geographically diverse sample, including both urban and rural areas. In the second stage, one adult per household is selected using the Next Birthday Method to minimize bias. Additionally, HINTS-6 oversampled high minority populations and rural areas, which have historically been underrepresented in health research. This approach helps ensure that the findings are reflective of both urban and rural populations and more accurately capture the diverse health concerns of racial and ethnic minorities.

### 2.3. Sample Size and Statistical Power

HINTS-6 aimed to collect 7000 completed surveys, which provides a large enough sample to ensure statistical power for detecting meaningful relationships between telehealth visit reasons and perceived discrimination. Based on our specific sample, we isolated 6017 participants who reported on discrimination inquiry. The survey’s sample size is considered large enough to detect small to moderate effect sizes, crucial for public health research. The oversampling of minority and rural populations bolsters the statistical power by ensuring adequate representation of historically marginalized groups. This sampling strategy is particularly important for understanding how demographic and socio-economic factors influence health outcomes and discrimination, ensuring the generalizability of findings across various population groups.

### 2.4. Data Collection

HINTS-6 utilized a mixed-mode data collection strategy to increase participation across diverse populations. Respondents had the option to complete the survey online or on paper, which was designed to maximize accessibility, particularly for individuals with limited internet access. To further enhance response rates, additional mailings and incentives were offered to non-respondents. Participants were given a USD2 prepaid monetary incentive, with further bonuses for completing the survey online. The data collection period ran from 7 March 2022 to 8 November 2022. This approach aimed to increase participation rates, particularly among hard-to-reach groups, and ensure a representative sample for this study.

### 2.5. Measures

The primary dependent variable in this study is self-reported discrimination in healthcare, which was assessed with the question: “Have you ever been treated unfairly or been discriminated against when getting medical care because of your race or ethnicity?” Responses were dichotomized into “Yes” or “No.”

The main independent variable is the reason for the most recent telehealth visit, categorized into four groups: “Annual Visit”, “Minor illness/Acute care”, “Chronic disease management”, and “Medical Emergency/Mental Health & Substance use”.

Several socio-demographic variables were included as covariates, including age, sex, education, income, and marital status. The confounding variables include: Sex: Respondents were asked, “On your original birth certificate, were you listed as male or female?” Sexual Identity: “Do you see yourself as…?” with options including “Heterosexual” and “Not Heterosexual.” Employment Status—“Which of the following best describes your current occupational status?” Marital Status: “What is your marital status?” with categories “Married,” “Not Married,” and “Others.” Educational Attainment was Categorized as “<Post High School,” “Some College,” “College Graduate,” or “Postgraduate.” Income was categorized using the Poverty Income Ratio (PIR), which reflects annual income relative to household size and the cost of living. PIR was used to classify income as low income (PIR < 1), middle income (1 ≤ PIR < 4), and high income (PIR ≥ 4), with a dichotomized cutoff at PIR = 1 for analysis.

### 2.6. Data Analytic Plan

Descriptive statistics was computed to summarize the socio-demographic characteristics of the study population and the reasons for their most recent telehealth visits. Bivariate analyses (chi-square tests or *t*-tests) was used to examine the relationship between telehealth visit reasons and perceived discrimination in care, while controlling for socio-demographic covariates. Next, logistic regression models were employed to estimate the odds ratios (ORs) for experiencing discrimination based on the primary reason for telehealth visits. These models were adjusted for confounding variables such as age, sex, sexual identity, marital status, income, and educational attainment. Stratified analyses were conducted by race/ethnicity to further explore the association between telehealth visit reasons and perceived discrimination among different racial and ethnic groups. Adjusted odds ratios were reported with 95% confidence intervals (CIs). Statistical significance was set at *p* < 0.05. All analyses were performed using SAS 9.4 statistical software. Stratified analyses were also conducted by race/ethnicity to explore potential effect modification. Notably, no data was deleted in order to maintain statistical rigor.

## 3. Results

### 3.1. Socio-Demographic Characteristics of the Study Population

Table 1 presents the socio-demographic attributes of the study population (*n* = 6017). The mean age of participants was 55.73 years (±17.36). Women comprised 60.4% of the sample. Regarding employment, 50.94% of participants reported being employed. In terms of racial distribution, 57.7% of the sample identified as non-Hispanic White, 15.92% as non-Hispanic Black, 17.9% as Hispanic, and 8.51% as belonging to other racial groups. Marital status data showed that 51.5% of participants were married, 29.26% were single, and 19.23% were divorced or separated.

Educational attainment varied within the sample, with 32.05% having completed post-high school education, 21.38% having attended some college, 27.64% being college graduates, and 18.93% having completed postgraduate training. The majority of participants (92.21%) identified as heterosexual. Annual income distribution revealed that 43.58% of the sample reported earning less than USD50,000, 29.72% earned between USD50,000 and USD100,000, while 26.7% earned more than USD100,000. Discrimination in accessing services was reported by 8.13% of participants.

The most common reasons for the most recent telehealth visits included annual check-ups (19.96%), acute care-related concerns (25.11%), chronic disease management (24.38%), and mental health, medical emergencies, behavioral health, or substance use-related concerns (30.55%).

### 3.2. Socio-Demographic Characteristics by Discrimination Status

Table 2 presents socio-demographic characteristics stratified by reported experiences of discrimination. Among individuals who endorsed experiencing discrimination, 65.2% were male. A higher proportion of individuals in this group were employed (53.83%). Racial distribution among those who reported discrimination showed that 18.94% were non-Hispanic White, 41.80% were non-Hispanic Black, 26.33% were Hispanic, and 12.93% identified as belonging to other racial groups. Marital status data indicated that 42.11% were married, 28.73% were single, and 29.17% were divorced or separated. Among those who reported discrimination, 89.64% identified as heterosexual. Income distribution within this group indicated that 51.36% earned less than USD50,000 annually, 29.55% earned between USD50,000 and USD99,000, and 19.09% earned more than USD100,000. Regarding the purpose of the most recent telehealth visits among those who experienced discrimination, 17.92% were for annual check-ups, 20.28% were for minor or acute illness, 31.6% were for chronic disease management, and 30.19% were for mental health, behavioral health, or substance use-related concerns. See Table 3 for the sociodemographic distribution based on the reason for the recent telehealth visit.

### 3.3. Association Between Socio-Demographic Characteristics and Perceived Discrimination in Care

Table 3 presents the crude and adjusted odds ratios (ORs) examining the association between socio-demographic characteristics and self-reported discrimination in care. In the unadjusted analysis, age was inversely associated with discrimination (crude OR: 0.98, 95% CI: 0.975–0.986). Males were more likely than females to report discrimination (crude OR: 1.525, 95% CI: 1.083–2.146). Regarding racial differences, non-Hispanic Black individuals had significantly higher odds of reporting discrimination compared to non-Hispanic White individuals (crude OR: 11.85, 95% CI: 7.67–18.309). Similarly, Hispanic individuals (crude OR: 4.626, 95% CI: 2.899–7.384) and individuals of other racial backgrounds (crude OR: 6.883, 95% CI: 4.04–11.729) had significantly increased odds of experiencing discrimination. Marital status was also associated with discrimination, with divorced or separated individuals having greater odds compared to those who were married (crude OR: 1.694, 95% CI: 1.158–2.478). Income was another significant factor; individuals earning less than USD50,000 per year had increased odds of discrimination compared to those earning more than USD100,000 (crude OR: 1.839, 95% CI: 1.261–2.683). No statistically significant association was found between the primary reason for the most recent telehealth visit and discrimination in the crude analysis.

In the adjusted analysis, the association between age and discrimination remained significant (adjusted OR: 0.974, 95% CI: 0.962–0.987). Racial disparities persisted, with non-Hispanic Black individuals experiencing the highest adjusted odds of discrimination (adjusted OR: 12.227, 95% CI: 7.711–19.387), followed by Hispanic individuals (adjusted OR: 3.761, 95% CI: 2.310–6.122) and individuals of other racial backgrounds (adjusted OR: 5.928, 95% CI: 3.438–10.222).

The primary reason for telehealth visits was significantly associated with perceived discrimination. Specifically, individuals utilizing telehealth for chronic disease management had increased odds of reporting discrimination compared to those using it for annual visits (adjusted OR: 2.001, 95% CI: 1.213–3.300). However, no statistically significant differences were observed for individuals using telehealth for acute care (adjusted OR: 1.22, 95% CI: 0.709–2.1) or for mental health and substance use disorder treatment (adjusted OR: 1.379, 95% CI: 0.827–2.299). Interaction terms were constructed to examine the relationship between discrimination and utilization rates of telehealth services; however, no statistically significant associations were observed. To explore the clinical relevance of reasons for using telehealth services, subgroup analyses based on gender, income, marital status, and educational attainment similarly revealed no significant interactions. Notably, race emerged as the only variable demonstrating a statistically significant relationship with telehealth-related discrimination.

### 3.4. Sub-Analysis: Race and Discrimination by Primary Reason for Telehealth Use

Table 4 presents the stratified analysis of the association between race and perceived discrimination based on the primary reason for the most recent telehealth visit. Among those using telehealth for annual visits, non-Hispanic Black individuals had significantly increased odds of reporting discrimination compared to non-Hispanic White individuals (OR: 16.388, 95% CI: 4.686–57.312). Similarly, Hispanic individuals (OR: 6.098, 95% CI: 1.412–26.341) and individuals of other racial backgrounds (OR: 6.687, 95% CI: 1.288–34.718) reported higher odds of discrimination. For individuals using telehealth for acute care, non-Hispanic Black individuals had an OR of 14.722 (95% CI: 6.013–36.044), indicating a significantly higher likelihood of perceived discrimination compared to non-Hispanic White individuals. Hispanic individuals had an OR of 3.347 (95% CI: 1.292–8.672), and those of other racial backgrounds had an OR of 4.417 (95% CI: 1.410–13.832). Among individuals using telehealth for chronic disease management, non-Hispanic Black individuals again reported significantly increased odds of discrimination (OR: 11.253, 95% CI: 4.927–25.699), followed by Hispanic individuals (OR: 5.705, 95% CI: 2.404–13.536) and individuals of other racial backgrounds (OR: 10.47, 95% CI: 4.111–26.672). See Appendix A for Table A1 showing the frequency distribution by primary reason for telehealth use.

Finally, for those using telehealth for mental health or substance use disorder treatment, non-Hispanic Black individuals had an OR of 11.143 (95% CI: 5.119–24.257), Hispanic individuals had an OR of 4.399 (95% CI: 1.998–9.684), and individuals of other racial backgrounds had an OR of 6.376 (95% CI: 2.457–16.544), all indicating significantly increased odds of discrimination compared to non-Hispanic White individuals. These findings highlight significant racial disparities in perceived discrimination during telehealth visits, with non-Hispanic Black individuals consistently reporting the highest odds of discrimination across all categories of telehealth use.

## 4. Discussion

This study found considerable socio-demographic characteristics that were associated with perceptions of discrimination in telehealth care. The sample size of the study cohort was 6017; the mean age was 55.73 years; and women (60.4%) outnumbered men. Through their socio-demographic profile, the bulk of the population were non-Hispanic White, earned under USD50,000 a year and were employed. This finding is supported by literature that highlights significant race/ethnicity-related biases in the measurement of Negative Parenting and treatment outcomes [29]. Of the sample, 8.13% reported discrimination in accessing services, a significant aspect of healthcare inequities in telehealth. Socio-demographic features and perceived discrimination segmenting the data by discrimination status shows important disparities. A significantly higher proportion of those who experienced discrimination were males (65.2%) and in employment (53.83%). Racial differences were particularly pronounced, with non-Hispanic Black, Hispanic, and members of other racial/ethnic groups significantly more likely to report discrimination experiences. The racial differences are consistent with prior documentation of disparities in the processes of care in healthcare settings, with minority groups consistently having greater challenges to equitable care [15]. Similarly, exploring data on income and marital status helps reinforce the socioeconomic impact on healthcare as a factor to be considered in evaluating healthcare outcomes. People with lower incomes, particularly those who made less than USD50,000 a year, were much more likely to say they had experienced discrimination. Though this relationship did not show a statistically significant relationship after adjustment for covariates. Notably, the main reasons for telehealth visits were also different for people who experienced discrimination. The majority of telehealth visits were for chronic disease management and mental health or substance use issues, underscoring the importance of paying close attention to vulnerable populations, who are more prone to experiencing care disparities [31]. Given that a significant percentage of individuals are using telehealth for behavioral health and substance use-related issues, this indicates that there is substantial overlap between barriers to accessing healthcare and barriers to mental health or substance use services.

This study’s findings provide important insights into the factors associated with perceptions of discrimination in telehealth care. Unadjusted analysis detected differences by age, sex, and race. Non-Hispanic Black subjects reported the highest odds of discrimination, which aligns with existing literature highlighting racial discrimination in healthcare [32]. The relationship between marital status and discrimination, with divorced or separated individuals facing higher odds, deserves more attention in future studies, as it may indicate an accumulation of vulnerabilities, whether from a lack of social connection or not. The adjusted analysis showed that age, race, and income were principal correlates of perceived discrimination. The highest odds of discrimination were found in Non-Hispanic Black individuals, followed by Hispanic individuals, and the rest of the racial/ethnic groups. This further supports the results seen in studies of racial disparities in healthcare experiences [18,33]. Interestingly, patients reporting telehealth use for chronic disease management also had higher odds of discrimination, indicating that having a pre-existing health condition may be a significant predictor of experiencing discrimination, as those with chronic conditions tend to engage with the healthcare system closer in time to situations that expose them to interpersonal discrimination. Though there was no statistically significant relationship in terms of discrimination with mental health and SUD treatment, however may be an attributable factor as well as mental health and SUD treatment are chronic in nature [34]. Thus, further research is needed. Reported research supports these findings, showing that individuals with chronic obstructive pulmonary disease and heart failure are less likely to use telehealth compared to those with diabetes, due to perceived fear of being discriminated [35]. As telehealth remains an ongoing healthcare delivery option, it is recommended that strategies be implemented to educate and provide resources aimed at promoting equity for Non-Hispanic Black patients. Finally, the results underscore the importance of the investigation of discrimination in the context of telehealth use, given the continued disparities along multiple demographic dimensions. Notably, racial and ethnic minorities especially non-Hispanic Black, Hispanic, and other racially minoritized groups exhibited consistently elevated odds of perceived discrimination across various telehealth visit types, highlighting systemic inequities in digital healthcare encounters.

### 4.1. Implications for Mental Health, Substance Use, and Public Health Practice

Our study findings have wide implications, such as for the practices of mental health and substance use care, but also extend to public health strategies more generally. Telehealth has become an essential means of enhancing access to healthcare services, especially since the midst of the COVID-19 pandemic [36]. While there was no specific contextual information about these populations, the observed racial and socio-economic disparities in this study stresses the need to rapidly adapt policies to ensure telehealth is not widening the inequality gap. These disparities are particularly apparent for mental health and substance use disorder treatment services, in which those who reported discrimination were highly engaged. These disparities should be on the radar of policymakers and health systems to address possible gaps in technology, cultural competency, and the delivery of care. In mental health and substance use care, where stigma and discrimination already pose major problems, the additional perception of discrimination experienced during telehealth visits may compound the issue and contribute to individuals forgoing or disengaging from care [37]. Thus, this suggests that experiences of discrimination, particularly among racially minoritized groups and those managing chronic conditions, may erode trust in telehealth as a reliable and equitable mode of healthcare delivery. Such diminished trust can hinder engagement with healthcare systems more broadly, reinforcing existing disparities in access, continuity of care, and health outcomes [2]. However, to ensure equitable access to care, we will need a multi-faceted approach that involves not only training healthcare providers on the importance of cultural competency, implementing policies that redacted socio-economic barriers to telehealth, but also advocating for greater resources towards supporting marginalized communities. This warrants the need to address these barriers through public health initiatives and inclusive policies to facilitate equitable access to healthcare for all, regardless of racial background or income level.

### 4.2. Impact on Positive Social Change

The insights gained from this research are invaluable to promoting social change by drawing attention to the ongoing racial inequities in healthcare, especially concerning telehealth. This study calls for systemic changes that look to minimize discrimination and ensure that all individuals, especially those from underrepresented racial and socioeconomic backgrounds, are provided with equal access to telehealth resources. Additionally, the increased odds of discrimination among those using telehealth for chronic disease management suggest that structural barriers may be more pronounced in contexts requiring ongoing care, rather than one-time or episodic services. These trends underscore the importance of intersectional and tailored policy responses if digital health platforms are to be used to equitably serve all populations and prevent the exacerbation of existing health disparities. Furthermore, efforts towards better cultural competence of telehealth services and mitigating the impact of discrimination on health outcomes should be a priority for policymakers and healthcare providers. This also allows society to make strides towards a fairer healthcare system in which telehealth services are equally accessible in the telehealth world, regardless of someone’s situation.

### 4.3. Strengths and Limitations

One of the major strengths of this study is its large, diverse sample, a strength that facilitates generalizability across a wide range of socio-demographic groups. Additionally, the use of both crude and adjusted odds ratios in the analysis of the association between socio-demographic characteristics and perceived discrimination enhances the strength of the findings. And the stratified analysis by the reason for telehealth use provides insights into how discrimination might differ across different forms of care, which is new in healthcare research. However, certain limitations should be mentioned. These data reflect one time point, so causal relationships between socio-demographic factors and perceived discrimination cannot be determined, as this is a cross-sectional study. Furthermore, because the discrimination measure was self-reported, it is subject to recall bias and social desirability bias. Thus, respondents may underreport their experiences, or conversely overreport them out of fear of stigma or social pressures, perhaps. What is not provided in this study is any information regarding which healthcare providers or institutions that care were accessed, more specifically, to understand if strict systemic factors lay within the garnered levels of discrimination in care surrounding the institution. Finally, because the study specifically evaluated telehealth visits, it does not capture the potential for discrimination within in-person healthcare visits, which may have different characteristics or rates.

## 5. Conclusions

This study portrayed sociodemographic discrimination characteristics, especially race-based based among participants. These findings add to the growing literature on disparities in healthcare, especially in the area of telehealth. It highlights the importance of targeted policies and interventions to overcome the socio-demographic and racial disparities in access to care, notably in mental health and substance use services. Telehealth has the opportunity to increase access, but must do so in a manner that ensures equitable experiences for all patients. Hence, there is an urgent need for equity-focused telehealth policies and culturally responsive care models to rebuild trust and ensure inclusive access across all populations. Thus, only through a purposeful reduction in discrimination can we ultimately achieve healthcare equity and attain meaningful social change.

## Figures and Tables

**Table 1:** Characteristics of the HINTS-6 Population.

Characteristics	Sample (*n* = 6017)	
	*n*	%
Age (Mean ± SD)		55.73 ± 17.36
Gender		
Male	2279	39.59
Female	3477	60.41
Employment		
Yes	2933	50.94
No	2825	49.06
Race		
NHW	3167	57.70
NHB	874	15.92
Hisp	981	17.87
Others	467	8.51
Marital Status		
Married	2957	51.51
Not Married	1680	29.26
Divorced/Widowed/Separated	1104	19.23
Education		
<Post High School	1842	32.05
Some College	1229	21.38
College Graduate	1589	27.64
Postgraduate	1088	18.93
Sexual orientation		
Heterosexual	5173	92.21
Not Heterosexual	437	7.79
Annual Income		
<USD49,999	2375	43.58
USD50,000–USD99,999	1620	29.72
>USD100,000	1455	26.70
Telehealth		
Annual Visit	469	19.96
Minor/Acute Illness	590	25.11
Chronic Disease	573	24.38
Emergency/MH and SUD	718	30.55
Discrimination		
Yes	449	8.13
No	5528	91.87

MH and SUD—Mental Health and Substance Use Disorder; NHW—Non-Hispanic White; NHB—Non-Hispanic Black; Hisp—Hispanic.

**Table 2:** Sociodemographic attributes of HINTS-6 by Discrimination Status.

Characteristics	Discrimination		
	Yes	No	*p*-Value
	(%)	(%)	
Gender			<0.0001
Male	34.80	40.00	
Female	65.20	60.00	
Employment			<0.0001
Yes	53.83	50.69	
No	46.17	49.31	
Race			<0.0001
NHW	18.94	61.02	
NHB	41.80	13.71	
Hisp	26.33	17.15	
Others	12.93	8.13	
Marital Status			<0.0001
Married	42.11	52.32	
Not Married	28.73	29.31	
Divorced/Widowed/Separated	29.17	18.37	
Education			0.0003
<Post High School	1842	32.07	
Some College	1229	21.11	
College Graduate	1589	27.89	
Postgraduate	1088	18.93	
Sexual orientation			<0.0001
Heterosexual	89.64	92.43	
Not Heterosexual	10.36	7.57	
Annual Income			<0.0001
<USD49,999	51.36	42.89	
USD50,000–USD99,999	29.55	29.74	
>USD100,000	19.09	27.37	
Telehealth			0.0542
Annual Visit	17.92	20.16	
Minor/Acute Illness	20.28	25.58	
Chronic Disease	31.60	23.67	
Emergency/MH and SUD	30.19	30.59	

MH and SUD—Mental Health and Substance Use Disorder; NHW—Non-Hispanic White; NHB—Non-Hispanic Black; Hisp—Hispanic.

**Table 3:** Crude and Adjusted Estimates for relationship with Discrimination.

Characteristics	Odds Ratio	
	Crude (95% CI)	Adjusted (95% CI)
Age (Mean ± SD)	0.980 (0.975–0.986)	0.974 (0.962–0.987)
Gender		
Male	1.525 (1.083–2.146)	1.151 (0.793–1.671)
Female	Ref	Ref
Employment		
Yes	0.907 (0.667–1.234)	0.739 (0.503–1.086)
No	Ref	Ref
Race		
NHW	Ref	Ref
NHB	11.850 (7.670–18.309	12.227 (7.711–19.387)
Hisp	4.626 (2.899–7.384)	3.761 (2.310–6.122)
Others	6.883 (4.040–11.729)	5.928 (3.438–10.222)
Marital Status		
Married	Ref	Ref
Not Married	1.331 (0.922–1.922)	1.161 (0.748–1.804)
Divorced/Widowed/Separated	1.694 (1.158–2.478)	0.830 (0.530–1.300)
Education		
<Post High School	1.247 (0.809–1.924)	0.718 (0.424–1.216)
Some College	1.206 (0.766–1.900)	0.710 (0.422–1.197)
College Graduate	0.888 (0.574–1.374)	0.670 (0.416–1.079)
Postgraduate	Ref	Ref
Sexual orientation		
Heterosexual	Ref	Ref
Not Heterosexual	1.170 (0.717–1.910)	1.097 (0.639–1.882)
Annual Income		
<USD49,999	1.839 (1.261–2.683)	1.273 (0.771–2.099)
USD50,000—USD99,999	1.145 (0.745–1.758)	1.010 (0.631–1.618)
>USD100,000	Ref	Ref
Telehealth Reason		
Annual visit	Ref	Ref
Acute Care	0.930 (0.563–1.537)	1.220 (0.709–2.100)
Chronic Disease	1.588 (0.995–2.535)	2.001 (1.213–3.300)
Emergency/MH and SUD	1.132 (0.707–1.812)	1.379 (0.827–2.299)

MH and SUD—Mental Health and Substance Use Disorder; NHW—Non-Hispanic White; NHB—Non-Hispanic Black; Hisp—Hispanic.

**Table 4:** Association between racial identification and discrimination for services by telehealth reason.

Characteristics	Odds Ratio (95% CI)			
	Annual Visit (*n* = 469)	Acute Care (*n* = 590)	Chronic Disease (*n* = 573)	Emergency/MH and SUD (*n* = 718)
Race				
NHW	Ref	Ref	Ref	Ref
NHB	16.388 (4.686–57.312)	14.722 (6.013–36.044)	11.253 (4.927–25.699)	11.143 (5.119–24.257)
Hisp	6.098 (1.412–26.341)	3.347 (1.292–8.672)	5.705 (2.404–13.536)	4.399 (1.998–9.684)
Others	6.687 (1.288–34.718)	4.417 (1.410–26.672)	10.470 (4.110–26.672)	6.376 (2.457–16.544)

N/B—Gender, Income, Marital status, and Educational attainment were not statistically significant for Telehealth reasons. MH and SUD—Mental Health and Substance Use Disorder; NHW—Non-Hispanic White; NHB—Non-Hispanic Black; Hisp—Hispanic.

## Data Availability

https://hints.cancer.gov/data/download-data.aspx accessed on 19 January 2025.

## Appendix A

**Table A1:** Sociodemographic attributes of HINTS-6 by Telehealth Reason.

Characteristics	Telehealth				
	Annual Visit	Acute Illness	Chronic Disease	Emergency/MH&SUD	*p*-Value
	*n* (%)	*n* (%)	*n* (%)	*n* (%)	
Gender					0.0006
Male	41.76	34.41	37.00	29.99	
Female	58.24	65.59	63.00	70.01	
Employment					<0.0001
Yes	42.92	66.31	41.32	55.59	
No	57.08	33.69	58.68	44.41	
Race					<0.0001
NHW	52.30	61.61	55.85	60.67	
NHB	22.28	10.05	15.85	11.95	
Hisp	16.95	19.74	19.62	19.67	
Others	8.47	8.59	8.68	7.72	
Marital Status					<0.0001
Married	50.56	62.84	55.68	49.71	
Not Married	29.35	19.75	29.30	28.15	
Divorced/Widowed/Separated	20.09	17.41	15.02	22.14	
Education					0.0009
<Post High School	32.51	21.22	27.79	23.43	
Some College	20.99	19.42	20.84	21.96	
College Graduate	26.86	31.83	28.34	33.24	
Postgraduate	19.64	27.52	23.03	21.38	
Sexual orientation					<0.0001
Heterosexual	92.72	92.53	92.51	85.84	
Not Heterosexual	7.28	7.47	7.49	14.16	
Annual Income					<0.0001
<USD49,999	48.42	28.28	43.33	39.31	
USD50,000–USD99,999	25.06	30.87	29.98	30.12	
>USD100,000	26.52	40.85	26.69	30.57	
Discrimination					0.0542
Yes	8.10	7.29	11.69	8.91	
No	91.90	92.71	88.31	91.09	

MH and SUD—Mental Health and Substance Use Disorder; NHW—Non-Hispanic White; NHB—Non-Hispanic Black; Hisp—Hispanic.
